# Protocol for the characterization of autoproliferating B and T cells from persons with multiple sclerosis

**DOI:** 10.1016/j.xpro.2025.104267

**Published:** 2025-12-10

**Authors:** Yuhan Qiu, Zoe Marti, Ivan Jelcic, Roland Martin

**Affiliations:** 1 Institute of Experimental Immunology, University of Zurich, 8057 Zurich, Switzerland; 2 Cellerys AG, Schlieren, Switzerland; 3 Neuroimmunology and MS Research Section (NIMS), Neurology Clinic, University of Zurich, University Hospital Zurich, 8091 Zurich, Switzerland; 4 Center for Molecular Medicine, Department of Clinical Neurosciences, Karolinska Institutet, Stockholm, Sweden

**Keywords:** Cell biology, Cell culture, Cell isolation, Cell separation/fractionation, Flow cytometry, Immunology

## Abstract

Autoproliferation (AP) is the spontaneous *in vitro* activation and proliferation of B- and T cells in the absence of exogenous stimulation. Here, we present a protocol to examine AP using peripheral blood mononuclear cells (PBMCs) derived from persons with multiple sclerosis (pwMS). We describe steps for the isolation of autoproliferating (AP+) lymphocytes as well as the expansion of AP^+^ T cells for downstream analyses. We detail procedures for standard fluorescence-activated cell sorting (FACS) and cell culturing.

For complete details on the use and execution of this protocol, please refer to Jelcic et al.^1^^,^^2^

## Highlights

• Steps for CFSE labeling and cell culture to track lymphocyte division
• Steps for isolation of autoproliferating B and T cells for analysis
• Steps for *in vitro* T cell expansion

## Abstract (editor highlights)

Publisher’s note: Undertaking any experimental protocol requires adherence to local institutional guidelines for laboratory safety and ethics.

## Graphical abstract

**Figure:**
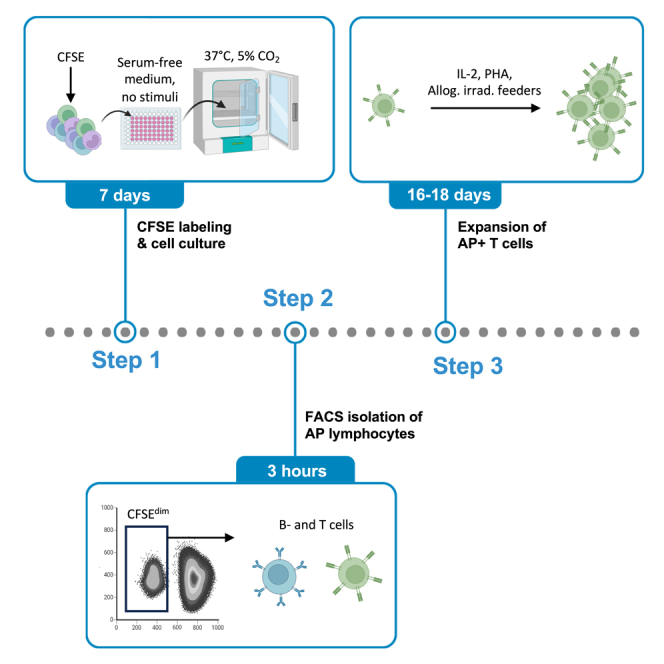

## Before you begin

Autoreactive lymphocytes are key contributors to multiple sclerosis (MS) pathogenesis.^1^^,^^2^^,^^3^ However, access to these pathogenic B- and T lymphocytes in MS is a critical limitation in investigating the disease. We have previously shown that in HLA-DR15+ MS individuals, these cells can proliferate without external stimuli *in vitro*, a process referred to as autoproliferation (AP). We also demonstrated that AP+ B- and T cells express proinflammatory (T-bet) and brain-homing (CXCR3) markers, form B-T cell-enriched clusters (BTECs),^2^ shown increased reactivity to self-peptides (AP+ T cells), secrete CXCL9 and IFN-γ, and can be found in the brain of pwMS. Conclusively, the AP+ compartment presents a promising and accessible source of pathogenic B- and T cells. Here, we present a protocol to access the AP+ lymphocyte compartment in PBMCs, which enables the study of AP+ lymphocytes. These characteristics point at the relevance of AP+ lymphocytes in the pathogenesis of MS. We describe steps for cell preparation, CFSE staining, co-culturing, and a flow cytometry-based assessment of AP. Our workflow permits sorting and downstream analysis of B cells as for example analysis for EBV infection, surface- and intracellular marker expression, transcriptional- and epigenetic profiling and B cell receptor repertoire studies.^2^ With respect to expansion, we have validated and described only the expansion of AP+ T cells for functional studies, as we lack experience with AP+ B-cell expansion.

### Innovation

The identification and isolation of pathogenic lymphocytes in MS is crucial for characterizing their phenotype, function, and antigen specificity. Both activated B- and T cells can migrate into the central nervous system (CNS) of pwMS, contributing to the initiation of autoreactive immune responses that ultimately cause irreversible tissue damage. To date, the most direct methods for identifying pathogenic lymphocytes have involved isolating them from CNS-derived samples such as cerebrospinal fluid (CSF) or lesion tissue or detecting peripheral lymphocytes with cross-reactivity to both MS-associated autoantigens and microbial antigens. However, CNS access is highly restricted, and immune cells are typically scarce within this compartment. Likewise, identifying cross-reactive lymphocytes in the periphery is technically challenging, low-throughput, and often yields only limited cell numbers.

Our approach allows for the simultaneous access to both B- and T lymphocytes within the AP+ compartment, and relies only on access to unmanipulated PBMCs samples from MS individuals. Therefore, the protocol introduces a novel and accessible strategy to enrich and characterize pathogenic human lymphocytes with improved efficiency and scalability.

### Institutional permissions

The described protocol requires access to human material. Approval from the responsible institutional ethics committee should be obtained prior to performing the experiment. In this protocol, signed informed consent was obtained from all patients and donors under a protocol approved by the Cantonal Ethics Committee (EC-No. 2014-0699, approved on 27 February 2015).

### Sample preparation

Before beginning with the protocol, the user needs to have access to PBMCs isolated from biosamples such as leukaphereses or whole blood. These can be obtained using standard protocols using density gradient centrifugation.^4^ The steps described below utilize cryopreserved PBMCs as the starting material.

## Key resources table

**Table:** 

REAGENT or RESOURCE	SOURCE	IDENTIFIER
**Antibodies**		

Anti-human CD3, 1:100 dilution	BioLegend	Cat# 317327
Anti-human CD4, 1:100 dilution	BioLegend	Cat# 317438
Anti-human CD8, 1:100 dilution	BioLegend	Cat# 344718
Anti-human CD19, 1:100 dilution	BioLegend	Cat# 363006

**Biological samples**		

Patient-derived peripheral blood mononuclear cells	–	N/A

**Chemicals, peptides, and recombinant proteins**		

CellTrace CFSE Cell Proliferation Kit	Thermo Fisher Scientific	Cat# C34570
Dimethyl sulfoxide (DMSO)	AppliChem	Cat# A3672
DNase I recombinant	Roche	Cat# 04536282001
Zombie NIR Fixable Viability Kit	BioLegend	Cat# 423105
NaN3	Sigma-Aldrich	Cat# S2002
Human IL-2 Recombinant Protein	Thermo Fisher Scientific	Cat# 200-02-100UG
PHA Purified	Thermo Fisher Scientific	Cat# R30852801

**Software and algorithms**		

FlowJo	TreeStar	https://www.flowjo.com/; RRID:SCR_008520
GraphPad Prism	GraphPad	http://www.graphpad.com/; RRID:SCR_002798

**Other**		

PBS	Thermo Fisher Scientific	Cat# 10010023
RPMI 1640 medium	Sigma-Aldrich	Cat# R0883
IMDM medium	GE Healthcare	Cat# SH30259.01
AIM-V medium	Thermo Fisher Scientific	Cat# 12055-091
Human serum	One Lambda	Cat# A25761
Fetal calf serum (FCS)	Eurobio	Cat# CVFSVF0001
LIVE/DEAD Fixable Aqua Dead Cell Stain Kit	Thermo Fisher Scientific	Cat# L34957
Gentamicin	Sigma-Aldrich	Cat# G1397
Penicillin/Streptomycin	Corning	Cat# 30-002-Cl
Human TruStain FcX	BioLegend	Cat# 422301
Rad Source RS-2000	Rad Source	N/A
Nalgene Rapid-Flow Disposable Filter Units	Thermo Fisher Scientific	Cat# 566-0020
96-Well Microplate, PS, U-Bottom	Greiner Bio-One	Cat# 650180

***Note:*** Equivalent reagents from other vendors, e.g. another serum-free, complete medium, should in principle not limit successful implementation of the method; however, it may require additional optimization.

## Materials and equipment

**Table:** Complete IMDM medium

Reagent	Final concentration	Amount
IMDM medium	N/A	469.5 mL
Heat-decomplemented human serum	5%	25 mL
Penicillin/Streptomycin	100 U/mL Penicillin, 100 μg/mL Streptomycin	5 mL
Gentamicin	50 μg/mL	500 μL
**Total**	**N/A**	**500 mL**

***Note:*** Heat-decomplementation is performed by incubation for 30 min at 56°C. Heat-decomplemented human serum and penicillin/streptomycin are stored at -20°C and thawed in a 37°C water bath prior to use. IMDM medium, PBS and gentamicin are stored at 4°C. After mixing all reagents, they need to be sterile filtered using a 0.2 μm filter. Complete IMDM medium can be stored at 4°C for up to 1 month.

**Table:** Wash solution

Reagent	Final concentration	Amount
PBS	1×	199.8 mL
Heat-decomplemented human serum	0.1%	200 μL
**Total**	**N/A**	**200 mL**

***Note:*** Store solution at 4°C for up to 2 months. After mixing all reagents, they need to be sterile filtered using a 0.2 μm filter.

**Table:** Quenching medium

Reagent	Final concentration	Amount
RPMI medium	N/A	180 mL
Heat-decomplemented human serum	10%	20 mL
**Total**	**N/A**	**200 mL**

***Note:*** After mixing all reagents, they need to be sterile filtered using a 0.2 μm filter. Store medium at 4°C for up to 2 months.

**Table:** FACS buffer

Reagent	Final concentration	Amount
PBS	1×	98 mL
Heat-decomplemented human serum	2%	2 mL
NaN_3_	0.02%	20mg
**Total**	**N/A**	**100 mL**

Store buffer at 4°C for up to two months.

## Step-by-step method details

**Figure 1:**
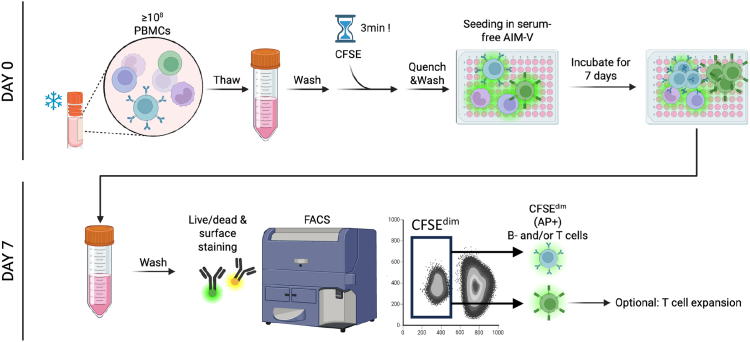
Detailed workflow on accessing AP+ B- and T lymphocytes from PBMCs of MS donors

The experimental outline was summarized in Figure 1.

### CFSE labeling and cell culture

**Timing: 3 h (hands-on) + 7 days cell culture**

The following steps are performed on day 0 and under sterile conditions. Before starting, cool down centrifuge to 4°C and load with buckets compatible with 50 mL falcon tubes.

1. Cell preparation
  a. Pre-warm complete IMDM medium to 37°C and transfer 20–30 mL into a 50 mL Falcon tube.
  b. Thaw the cryopreserved PBMCs by swirling the vial in 37°C water bath until almost fully thawed. Immediately transfer the cells into pre-warmed complete IMDM medium.
    ***Note:*** To obtain enough AP+ B/T cells for downstream application, it is recommended to start with ≥10^8^ PBMCs. Consider up to 50% of cells during freeze-thaw and another 25% loss of the initial cell counts during CFSE labeling (total cell loss: 75% of starting population).
  c. Centrifuge at 400 g for 5 min at 22°C.
  d. Discard the supernatant and resuspend the pellet with 20 ml wash solution.
    ***Note:*** When cell clumping is observed, centrifuge to obtain a pellet and resuspend the cells in serum-free AIM-V medium containing 50 U/mL DNase I. Incubate at 37 °C for 15 min.
  e. Centrifuge at 400 g for 5 min at 22°C.
  f. Discard the supernatant and resuspend the cells in 5 mL wash solution.
    i. Count the cells.
    ii. Adjust the concentration of cells to up to 10^7^ cells/mL. Keep the cell suspension in a 50 mL tube.

2. CFSE staining
  a. Turn off the light in the laminar flow hood.
  b. Reconstitute CellTrace CFSE immediately prior to use by adding the 18 μL of DMSO to one vial of CFSE dye. This yields a 5 mM stock solution.
    ***Note:*** It is important to protect the CFSE from light. Protect reagents and plates using aluminum foil throughout the experiment.
  c. Prepare the working solution by adding 2 μL of 5 mM CFSE stock to 10 mL of sterile PBS in a 15 mL Falcon tube. This yields a 1 μM working solution.
  d. Add CFSE working solution to the 50 mL tube containing the PBMC suspension at a ratio of 1:1 (e.g., mix 10 mL of cell suspension with 10 mL of CFSE working solution).
  e. Incubate the cells for exactly 3 min, gently swirling the suspension every ∼30 s.
    **CRITICAL:** The exact timing of this step is crucial and will significantly affect the viability of the obtained cells.
  f. Stop the CFSE labeling by diluting the CFSE-labeled PBMCs 1:5 in cold quenching medium (e.g., dilute 2 mL of CFSE-labeled cells by adding extra 10 mL quenching medium).
  g. Centrifuge at 400 g for 10 min at 4°C.
  h. Discard the supernatant and resuspend the pellet with 20 mL AIM-V (serum-free) medium.
  i. Centrifuge at 400 g for 5 min at 22°C.

3. Seeding and incubation
  a. Discard the supernatant and resuspend the cells in 5 mL AIM-V medium.
    i. Count the number of cells.
    ii. Adjust the concentration of the CFSE-labeled cells to 1×10^6^ cells/mL.
  b. Seed 200 μL of the cell suspension (2 × 10^5^ cells) per well in 96-well U-bottom plates.
    ***Note:*** Seed at least 10 replicate wells for each sample. We recommend using Greiner U-bottom plates. Do not use flat- or V-bottom plates. Avoid seeding cells in the outer rim of wells; instead, fill those wells with 200 μL of sterile PBS to minimize evaporation during incubation.
  c. Incubate the cells for 7 days at 37°C in a humidified incubator with 5% CO_2_.
    ***Note:*** We recommend covering the plates loosely with aluminum foil to shield them from light. Don’t wrap the plate to tight to allow air access to the cells. It also serves as a reminder not to expose the cells to light during the following steps. Make sure that the plate is not disturbed/shaken during the incubation time in order to avoid breaking up cell-to-cell contacts, which are critical to initiate the AP.

### AP lymphocyte isolation

**Timing: 3 h**

The following steps are performed on day 7 under sterile conditions. Protect the cells from light during the entire process.

4. Antibody staining
  a. After 7 days culture, remove the plates from the incubator to the biosafety cabinet.
  b. Carefully transfer 100 μL supernatant from each well to a new plate for cytokine analysis if desired. Make sure not to touch the cell pellet at the bottom.
  c. Resuspend the remaining cells and pool the replicates into a separate FACS tube.
    ***Note:*** Use sterile tubes if cells shall be used for further steps.
  d. Centrifuge at 400 g for 5 min at 4°C.
  e. Discard the supernatant and re-suspend the cells in 1 mL PBS.
  f. Centrifuge at 400 g for 5 min at 4°C.
  g. Prepare the FcR blocking and live/dead staining mix (PBS + 1:100 live/dead + 1:20 FcR blocking reagent). For each condition/tube, 100 μL of this staining mix will be needed. Calculate and prepare the reagents accounting for a 10% excess volume.
  h. Discard the supernatant and resuspend each sample in 100 μL FcR blocking and live/dead staining mix.
  i. Incubate for 30 min at 4°C.
  j. Wash the cell by adding 2 mL ice-cold FACS buffer.
  k. Centrifuge at 400 g for 5 min at 4°C.
  l. Prepare the antibody staining mix by diluting each antibody 1:100 in FACS buffer.
    ***Note:*** Per 10 replicates wells, 100 μL of staining mix will be needed.
  m. Add 100 μL antibody staining mix to each sample and vortex briefly.
  n. Incubate for 30 min at 4°C.
  o. Wash the cell by adding 2 mL cold FACS buffer.
  p. Centrifuge at 400 g for 5 min at 4°C.
  q. Resuspend in 500 μL cold FACS buffer and keep samples on ice while performing the compensation.
  r. Samples are read using a conventional or spectral flow cytometer.
    ***Note:*** CFSE^dim^ B- and T cell populations, i.e. cells that have undergone proliferation and therefore show CFSE dilution (Figure 2), can be sorted for downstream analysis or expansion.
    **Figure 2:**
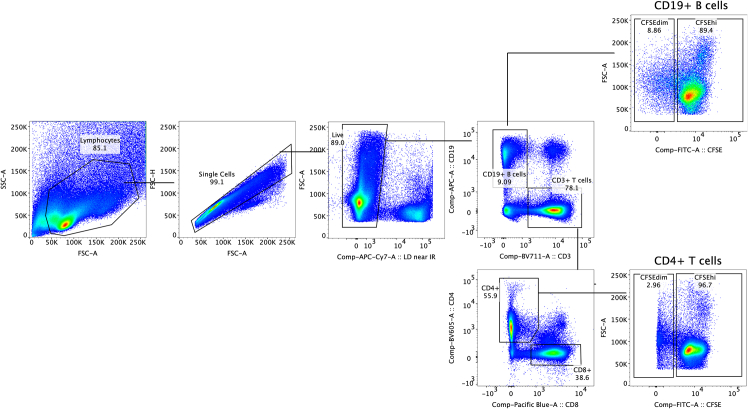
Representative flow cytometry results demonstrating autoproliferation of CD4+ and CD19+ B T cells in PBMCs from pwMS

### T cell expansion

**Timing: 30 min (hands-on) + 14–16 days cell culture**

The following steps are performed if AP+ CD4+ T (AP+ T) cells are sorted and need to be enumerated for downstream analyses. Note that, for one 96-well U bottom plate used for expansion, a total of 12 × 10^6^ allogeneic PBMCs and 1.2 ×10^6^ AP T cells are used.

5. Preparation and seeding of sorted AP+ T cells
  a. Pre-warm complete IMDM to 37°C in a water bath.
  b. Count the number of AP+ T cells available for expansion.
  c. Centrifuge the cells at 400 g for 5 min at 22°C.
  d. Resuspend the AP+ T cells in pre-warmed complete IMDM to a final concentration of 2 × 10^5^ cells/mL.
  e. Prepare the required number of 96-well U-bottom plates based on the number of AP+ T cells you want to expand.
    ***Note:*** Cells will be seeded at 2 × 10^3^–2 × 10^4^ per well).
  f. Fill the rim of outermost wells with 200 μL of sterile PBS to minimize evaporation.
  g. Seed 100 μL of the AP+ T cell suspension into each well.

6. Feeding
  a. Fill a 15 mL tube with 10 mL of pre-warmed complete IMDM.
  b. Thaw a cryopreserved vial of allogeneic PBMCs by swirling it in the 37°C water bath until almost completely thawed.
  c. Immediately transfer the cells into the 10 mL of warm complete IMDM in the tube.
  d. Centrifuge the cells at 400 g for 5 min at 4°C.
  e. Aspirate the supernatant carefully without disturbing the pellet and resuspend the PBMC pellet in 10 mL of complete IMDM.
  f. Count the cells.
    ***Note:*** Ensure you have ∼2× the number of PBMCs required that add on top of the T cells. You need to seed them at 2 × 10^5^ cells/well.
  g. Irradiate the PBMCs with total dose of 45 Gray (Gy).
  h. Centrifuge the irradiated PBMCs at 400 g for 5 min at 4°C.
  i. Count the irradiated PBMCs and adjust the concentration to 2 × 10^6^ cells/mL in complete IMDM.
  j. Add PHA and IL-2 to the irradiated PBMCs suspension at a concentration of:
    IL-2: 40U/mL
    PHA: 2μg/mL
    ***Note:*** These concentrations are double the desired final concentration used in the cell culture, as they will be diluted 1:1 when added to the AP+ T cells.
  k. Seed 100 μL of irradiated PBMCs suspension per well on top of the AP+ T cells.
    ***Note:*** Allogeneic irradiated PBMCs serve as feeder cells that provide trophic support and essential costimulatory signals to sustain T cell growth.^5^
  l. Incubate the plate for 4 days at 37°C with 5% CO_2_.
  m. Every 3–4 days, aspirate 100 μL of supernatant from each well, and top up with 100 μL of warm complete IMDM supplemented with 40U/mL IL-2.
    ***Note:*** During culture, no PHA is added. Repeat this step every 3–4 days until (and including) day 12.

7. Resting and freezing/restimulation
  a. After day 12, observe the T cells culture every day under the microscope.
    ***Note:*** Usually, cells will stop proliferating soon after this time, which is indicated by the formation of a single round cell pellet and a lack of change in medium color.
  b. On day 16, aspirate the supernatant and top up with 100 μL warm complete IMDM without IL-2. Continue the daily check of cell pellet.
    ***Note:*** Resting is indicated by compact, round pellets and the absence of proliferative clusters. Cells can be cryopreserved in heat-decomplemented FCS with 10% DMSO, or used for downstream analyses, or re-stimulated by repeating the expansion starting from step 5.a.

## Expected outcomes

This protocol provides detailed information for assessing AP+ lymphocytes of human PBMCs and to harvest autoproliferating, potentially pathogenic lymphocytes for further assessment. Since AP is enhanced in HLA-DR15+ pwMS, the most suitable samples for this protocol are obtained from these individuals, preferentially during remission or under natalizumab treatment, which prevent lymphocytes from migrating to the brain^6^ and mounts circulating memory and marginal zone B cells.^7^ As a guide, the AP cells are composed of roughly 30-40% T cells (CD4+>CD8+), 30% B cells and 30% other cells.^1^

## Limitations

Without defined antigenic stimulation, the frequency of CFSE^dim^ B- and T cells in PBMC co-cultures is typically low ranging from 0-10% of live lymphocytes. Based on our experience, approximately 30-50% of donors do not show clear autoproliferation.^1^^,^^3^ As a result, the yield of proliferating CFSE^dim^ cells is often insufficient for downstream applications such as single-cell RNA sequencing or functional assays. To overcome this limitation, a large starting number of PBMCs is needed or an expansion step as provided for AP+ CD4+ T cells above. Unfortunately, a standardized protocol for the expansion of AP+ B or CD8+ T cells is not established yet.

## Troubleshooting

### Problem 1

Drastic cell loss after CFSE labeling.

### Potential solution

Begin with at least 10^8^ PBMCs. Anticipate up to 75% cell loss from the sample thawing, multiple washing steps and CFSE staining. Furthermore, ensure that the CFSE labeling is timed correctly and does not exceed the stated 3 min.

### Problem 2

Poor CFSE labeling caused by light exposure.

### Potential solution

Protect CFSE from light throughout the staining process. Protect the 96-well plate with aluminum foil during incubation to maintain dye stability.

### Problem 3

No CFSE^dim^ population is observed.

### Potential solution

Immediately perform a FACS analysis after CFSE labeling to check labeling efficiency. During incubation, keep the plate undisturbed. B- and T cells form immunological synapses and subsequently grow in clusters, and disrupting these clusters stops their growth. However, as noted above, not all donors show autoproliferation, and hence, the lack of a CFSE^dim^ population may simply reflect the absence of AP in a given donor. To exclude technical artifacts occurred during the experimental setup, we recommend using a positive control (e.g., using PHA as an antigen-independent stimulus, or anti-CD2/3/28 activation beads to stimulate T cell proliferation) to verify that the lack of CFSE^dim^ cells is not caused by impaired cell integrity.

## Resource availability

### Lead contact

Further information and requests for resources and reagents should be directed to and will be fulfilled by the lead contact, Roland Martin (roland.martin@uzh.ch).

### Technical contact

Technical questions on executing this protocol should be directed to and will be answered by the technical contact, Yuhan Qiu (yuhan.qiu@uzh.ch).

### Materials availability

This study did not generate new unique reagents.

### Data and code availability

This study did not generate new data and code.

## Acknowledgments

This study was supported by 10.13039/100010663European Research Council grant ERC-2013-ADG 340733 to R.M. The authors thank the patients for their donation of biosamples. The graphical abstract and Figure 1 were created with Biorender.com.

## Author contributions

Y.Q., Z.M., I.J., and R.M. conceptualized the protocol. Y.Q., Z.M., and R.M. wrote the manuscript. Z.M. performed the experiments. All authors reviewed and approved the manuscript.

## Declaration of interests

The authors declare no competing interests.
